# Determinants of walking fitness in patients with heart failure attending cardiac rehabilitation

**DOI:** 10.1136/openhrt-2018-000866

**Published:** 2019-02-27

**Authors:** Patrick Doherty, Alexander Stephen Harrison, Rashed Hossain

**Affiliations:** 1 Department of Health Sciences, University of York, York, UK; 2 Cardiology department, York Teaching Hospitals NHS Foundation Trust, York, UK

**Keywords:** physical fitness, heart failure, cardiac rehabilitation

## Abstract

**Introduction:**

Patients with heart failure (HF) attending cardiac rehabilitation (CR) benefit in terms of improved quality of life, physical fitness and reduced hospital admissions. Too few patients with HF attend CR and little data exist on the characteristics of those who do especially in respect of physical fitness. This study evaluates the extent by which clinical and demographic factors determine walking fitness in patients with a primary diagnosis of HF.

**Methods:**

Clinical data from the British Heart Foundation National Audit of Cardiac Rehabilitation identified 1519 patients with HF who completed an incremental shuttle walk test (ISWT). Stepwise regression accounting for age, gender and multiple potential confounders assessed their contribution to total walking distance.

**Results:**

Mean age was 64.5 (SD 12.70) years with a range of ISWT distances across gender and associated comorbidities from 215 to 282 m. Walking distance reduced by 4.9 m for each year increase in age above mean age (p<0.001). After accounting for confounders, females walked 42.1 m less than males (p≤ 0.001). Pulmonary disease and the existence of depression was associated with a 39.3 and 52.2 m reduction in walking distance, respectively. Body mass index >30 was associated with 28.5 m reduction in walking distance (p<0.001). HF severity failed to improve the regression model fit or achieve significance in the analysis

**Conclusions:**

Age, gender and the presence of pulmonary disease or depression were highly significant factors in predicting walking fitness in patients with HF. The study also produced a set of reference values based on these four factors to aid the interpretation of walking fitness in patients with HF.

**Key messages**

**What is already known about this subject?**

- Patients with heart failure should be referred to cardiac rehabilitation; however, less than 20% of eligible patients access such services.
- Exercise training and physical activity have been shown to benefit patients, yet too few programmes assess fitness prior to starting rehabilitation.
- Little is known about which clinical and patient characteristics determine fitness.

**What does this study add?**

- This study is the first to determine the extent by which comorbidities determine walking fitness in patients with a primary diagnosis of heart failure.

**How might this impact on clinical practice?**

- These findings have led to the development of walking fitness reference values which can be used by clinicians and patients to understand their level of fitness and help set realistic goals as part of their cardiac rehabilitation programme and long-term self-management.

## Introduction

Cardiac rehabilitation (CR) for patients with coronary heart disease and those with heart failure (HF) is a clinically effective intervention that has yet to achieve optimal uptake in routine clinical practice.^1–3^ The stated ambition of NHS England is to improve uptake from 45% in 2014 to greater than 65% by 2020 which is an initiative that aligns with the National Institute for Health and Care Excellence guidance recommendations in the UK^4 5^ and international guidance.^6 7^ Cardiologists and cardiac nurses play a fundamental role in the early treatment and management of heart disease and they represent the primary source of referral to CR.^8^

National audit data from the UK^3^ show that uptake to CR is around 50% for patients with acute coronary syndrome and equivalent to one-third in European and American CR programmes.^9 10^ The situation is much worse for patients with HF where uptake is less than 20% of all eligible patients in the UK.^3 11^ Referral to CR for patients with HF has yet to become routine practice with most programmes already stretched by the sheer volume of patients attending through the conventional cardiology referral pathways.^3 12^

We hypothesise that one of the reasons so few patients with HF attend CR and many programmes are unable to recruit patients with HF is, in part, due to a perception that ‘exercise training and rehabilitation’ are at odds with a patient’s diagnosis of HF. There is an urgent need to create a more realistic view of what a patient with HF can achieve in terms of physical exercise and fitness. Although clinical trial data on HF exist suggesting what is possible in terms of maximal exercise capacity, obtained from cardiopulmonary exercise testing (CPET), this tends to be based on an exclusive population that are much younger, by as much as 11 years, and have fewer comorbidities^1^ compared with patients who attend routine practice CR.^3^

The incremental shuttle walk test (ISWT) is the most commonly used test of functional physical fitness in the UK.^3^ Although the ISWT does not represent a ‘criterion maximal test’, of exercise capacity it is a recommended submaximal surrogate measure of exercise capacity^13^ that is positively validated against CPET.^14^ Some studies have used the ISWT to investigate potential determinants of walking fitness in conventional cardiac patients^15 16^ identifying age, height, body mass index (BMI) and the presence of diabetes as significant predictors of distanced achieved. The New York Heart Association (NYHA) Functional Classification is an established symptom and function-aligned measure, classifying the extent of HF severity in patients, yet has not been investigated for its role in determining walking fitness as measured by the ISWT in HF.

This study aims to investigate and assess the strength of association between walking fitness and relevant patient demographics, risk factors, comorbidities and severity of HF. Our findings aim to create new knowledge to guide clinical decisions about the characteristics of patients with HF taking part in exercise-based CR.

## Methods

This study used a robust observational methodology to evaluate the potential contribution of individual patient characteristics in defining physical fitness in patients with HF attending a CR assessment.

### Data

The study used data from a routinely collected audit of CR, British Heart Foundation (BHF) and the National Audit of Cardiac Rehabilitation (NACR). The NACR collects data from CR programmes across the UK and has a 74% coverage for electronic data entry.^3^ The electronic data were acquired in a link-anonymised format from 224 programmes, which collect data on patient’s demographics, risk factors and baseline measures prior to starting CR. The data collection of patient information is covered by Section 251 approval which is reviewed by NHS Digital annually, the rationale for data collection was to improve the quality of CR service delivery for public benefit. Patients were included if they had an initiating event (primary diagnosis of HF) between 1 January 2013 and 30 October 2018. To account for potential reporting bias through missing data, the HF population without an ISWT score was compared with the ISWT group in the context of age and gender. A planned subanalysis investigated the extent by which HF severity, defined by the NYHA Functional Classification, determined walking fitness.

The primary variable of interest (dependant variable) is maximum distance walked in metres measured by the ISWT as part of a prerehabilitation assessment. The ISWT is an externally paced (via audio player) and graded walk test with 12 levels of speed that has been validated in cardiac and pulmonary rehabilitation populations.^14 17^ Although the test result can be reported by the speed level achieved our planned analysis used distance walked as a continuous variable in the linear regression model which also enabled us to pursue reference values using a measure (metres walked) which are more relevant to clinicians and patients.

### Statistical analysis

The analyses were conducted in IBM statistical package SPSS V.25 (SPSS). Correlation and group comparisons used t-tests and Pearson correlation, respectively. Subject to having sufficient data to fulfil statistical distribution assessments (n>30) all potential covariates were investigated in the analysis. Backwards stepwise linear regression models were built to investigate whether, accounting for covariates, the patient-level factors were associated with walking fitness as measured by ISWT distance.

Relevant important covariates were included in the analysis, where they were evidenced in the literature or significant in preliminary analysis. Age (years), gender (male/female), marital status (single/not) and employment status have been shown to influence the outcomes following a variety of different rehabilitation interventions, including CR. Employment status was coded as employed/retired or unemployed, this is because previous research found that employed and retired states have similar effects on outcomes. Other risk factors such as BMI, physical activity, smoking status, psychosocial well-being measured through Hospital Anxiety Depression Scale (HADS) score and comorbidity were included as they are routinely reported by the NACR as variables that influence patient engagement with CR.^3 8^

Statistical level for significance was p<0.05 and actual significant values were expressed as reported up to 0.001. Data model checking was performed to ensure that the models were a good fit through assumptions associated with the regressions.

## Results

This study consisted of 1519 patients (68% male) with HF who had completed an ISWT. The mean age for total population was 64.5 (SD 12.7) years. Table 1 shows the average ISWT distances in metres for each of the included variables. The overall mean distance was 266.6 m (156.4 SD). Pearson correlation indicates that there is a significant negative relationship between age and ISWT distance of −0.40 (p≤0.001), which was stronger for females (r=−0.436) than males (r=−0.391).

**Table 1:** Incremental shuttle walk test (ISWT) distance for all included variables

Patient characteristics	ISWT distance (m)					
	Mean	SD	Count	%	Pearson correlation (PC)/mean difference (MD)/F stat ANOVA (F)	P value
Age	64.51	12.70	1519	−0.40 (PC)	<0.001
Gender						
Male	284.3	162.65	1035	68	55.71 (MD)	<0.001
Female	228.6	134.94	482	32		
Ethnicity						
White	263.7	151.47	1136	75	−11.74 (MD)	0.204
Non-white	275.4	170.15	383	25		
Smoking Status						
No	266.5	153.31	1304	93	−10.61 (MD)	0.503
Yes	277.1	179.31	104	7		
Physical activity status						
No	246.5	153.92	887	68	−60.85 (MD)	<0.001
Yes	307.4	149.56	408	32		
Body mass index (BMI)						
<30	276.2	163.18	827	59	25.42 (MD)	0.002
>30	250.8	138.64	586	41		
Employment status						
Employed	262.5	152.63	995	79	−9.53 (MD)	0.366
Unemployed	272.1	156.78	266	21		
Marital status						
Single	246.9	157.08	359	31	−19.36 (MD)	0.039
Partner	266.2	143.44	799	69		
HADS score: Anxiety						
Not anxious	271.6	155.81	1042	83	36.79 (MD)	0.002
Anxious	234.8	145.82	209	17		
HADS score: Depression						
Not depressed	274.2	154.71	1082	86	64.78 (MD)	<0.001
Depressed	209.4	143.38	169	14		
IMD score						
Lowest quintile	265.5	173.68	263	22.1	0.43 (F)	0.789
Second quintile	256.6	163.18	277	23.3		
Third quintile	259.6	144.28	221	18.6		
Fourth quintile	267.9	157.85	232	19.5		
Fifth quintile	274.3	161.18	196	16.5		
Total population ISWT score	266.6	156.41				

ANOVA, analysis of variance; HADS, Hospital Anxiety Depression Scale; IMD, Index of Multiple Deprivation; MD, mean difference; PC, pearson correlation.

In absolute terms males had a significantly larger ISWT distance on average 55.7 m greater than females (p≤0.001). Patients with a history of achieving moderate physically active status had on average a statistically significant 60.9 m greater distance than those who were not (p≤0.001). Patients with BMI greater than 30 demonstrated shorter ISWT distances by on average 25.4 m (p=0.002). Marital status of patients was significantly associated with walking fitness, those with a partner had 19.4 m greater than those single (p=0.039). No other variables were associated with differences in ISWT distance. Patients who were either anxious or depressed at baseline, HADS score ≥8, had significantly lower walking distances than those patients with HADS score below 8 (p<0.05).

Additionally, the study included as subset analysis the inclusion of NYHA class I–IV, the analysis failed to find any significant differences between the NYHA class and ISWT distance (p>0.05).

Table 2 shows the ISWT distance, when analysed against the patient’s comorbidity status. Eighteen comorbidities captured on the NACR were included, along with a single-variable coding multimorbidity of ≤3 or 3+. The results showed that patients having angina, arthritis, diabetes, stroke, hypertension, chronic obstructive pulmonary disease (COPD), asthma and chronic back problems demonstrated significantly lower ISWT distance (mean difference range 28.2–55.3, p<0.05).

**Table 2:** Incremental shuttle walk test (ISWT) distance reported by comorbidity category

	ISWT distance (m)					
	Mean	SD	Count	%	Mean difference	P value
Angina						
No	270.0	158.5	1419	93	51.6	0.001
Yes	218.5	114.1	110	7		
Arthritis						
No	276.3	158.3	1262	83	57.1	<0.001
Yes	219.2	137.5	257	17		
Cancer						
No	268.6	158.7	1383	91	22.0	0.117
Yes	246.6	130.2	136	9		
Diabetes						
No	276.7	161.1	1207	79	48.8	<0.001
Yes	227.9	129.9	312	21		
Stroke						
No	270.0	158.0	1424	94	53.1	0.001
Yes	216.9	120.6	95	6%		
Hypertension						
No	276.6	164.1	982	65	28.2	0.001
Yes	248.4	139.6	537	35		
Chronic obstructive pulmonary disease						
No	270.6	157.8	1409	93	55.3	0.001
Yes	215.4	126.9	110	7		
Asthma						
No	269.2	157.8	1394	92	31.4	0.031
Yes	237.8	137.1	125	8		
Chronic back problems						
No	272.0	158.5	1306	86	38.1	0.001
Yes	233.8	138.7	213	14		
Anxiety						
No	265.8	156.0	1402	92	−11.4	0.449
Yes	277.2	161.9	117	8		
Depression						
No	268.3	158.8	1372	90	16.8	0.217
Yes	251.5	131.4	147	10		
Family history						
No	266.0	159.1	1253	82	−3.5	0.741
Yes	269.5	143.1	266	18		
Erectile dysfunction						
No	267.3	157.4	1436	95	12.9	0.466
Yes	254.5	138.5	83	5		
Hypercholesterolaemia/dislipidaemia						
No	269.0	159.0	1280	84	15.3	0.166
Yes	253.8	141.7	239	16		
Comorbidities grouped						
≤3	282.0	166.0	967	64	42.2	<0.001
>3	239.8	134.1	552	36		

Osteoporosis, claudication and rheumatism were removed due to insufficient subset sample sizes (n≤25).

The number of comorbidities was also significant with a 42.19 m reduced mean difference with having more than three comorbidities.

Table 3 shows the results from the linear regression evaluating the association between ISWT distance against patient characteristics and related risk factors. The model confirms that age, after accounting for multiple potential confounders, was negatively associated ISWT distance (B=−4.868, p<0.001). The effect of age is centralised around the mean suggesting that for each single-year increase in age, above the mean, there is an associated 5.5 m reduction in distance walked.

**Table 3:** Linear regression findings for the incremental shuttle walk test (ISWT) by patient characteristics

Patient characteristics	B	SE	t	Significance	95% CI
Gender (Female)	−42.123	10.658	−3.952	<0.001	−63.055 to −21.191
Age	−4.868	0.447	−10.888	<0.001	−5.746 to −3.990
Ethnicity (Non-white)	−23.347	13.340	−1.750	0.081	−49.545 to 2.852
Physical activity status (150 min/week)	43.467	10.543	4.123	<0.001	22.761 to 64.173
BMI (>30)	−28.645	10.203	−2.808	0.005	−48.682 to −8.607
Employment status (Unemployed)	−50.336	12.951	−3.887	<0.001	−75.771 to −24.901
Diabetes	−33.448	12.318	−2.715	0.007	−57.639 to −9.257
Chronic back problems (Yes)	−36.855	13.392	−2.752	0.006	−63.155 to −10.554
COPD (Yes)	−39.310	18.937	−2.076	0.038	−76.501 to −2.119
HADS score: Depression (Depressed)	−52.194	14.842	−3.517	<0.001	−81.342 to −23.045
IMD score	11.624	3.801	3.058	0.002	4.160 to 19.088
Intercept	276.703	13.339	20.744	<0.001	250.506 to 302.899

R=0.530. R^2^=0.281. Adj R^2^=0.266.

BMI, body mass index; COPD, chronic obstructive pulmonary disease; HADS, Hospital Anxiety Depression Scale; IMD, Index of Multiple Deprivation.

Gender plays a significant part in determining walking fitness with female patients having a 42 m reduced walking distance (p<0.001). Other covariates of statistical significance associated were ethnicity, employment status, marital status, physical activity, BMI and socioeconomic class. Patients being unemployed, with greater BMI and not achieving physical activity status were all associated with a lower ISWT score between 29 and 50 m (p=0.05–0.001). For every increase in socioeconomic class quintile the patient’s baseline ISWT increased by 8.5 m, thus being from the lowest as opposed to the highest quintiles was a difference of 44 m (p=0.002). Patients being identified as clinically or borderline depressed at baseline using the HADS score performed on average 52 m worse than those who were classified as normal (p<0.001).

Variables that were not significant such as smoking status, other covariates and multimorbidity were automatically removed from the backward stepwise analysis.

The model was of good fit and the residuals met the assumptions of uniform variance and linearity, and the adjusted r^2^ value was 0.266. The grouping of comorbidities into less than or greater than 3 was not significantly associated with walking fitness. Subset analysis showed that the model with NYHA was not of greater fit. The inclusion of NYHA class was not statistically significant.

Table 4 presents our proposed reference values for ISWT distance split by age, gender and COPD set within 5, 25, 75 and 95 percentiles.

**Table 4:** Incremental shuttle walk test reference values for patients with heart failure (HF) split by age, gender and presence of chronic obstructive pulmonary disease (COPD) and depression

Heart failure (HF)+comorbidity category	Age and gender	Incremental shuttle walk test (ISWT)						Count
		Mean distance (m)	SD	Percentile 05	Percentile 25	Percentile 75	Percentile 95	
HF only	<67 years							
	Male	338	180	70	200	460	630	443
	Female	285	145	70	190	350	520	190
	67+ years							
	Male	243	138	60	140	330	480	434
	Female	184	109	40	100	250	390	211
HF+COPD	<67 years	237	133	40	120	330	430	51
	67+ years	197	120	40	90	270	470	59
HF+Depression	<67 years	261	143	30	150	340	520	95
	67+ years	223	107	70	155	295	420	52

## Discussion

Reassuringly our findings showed that patients with a primary diagnosis of HF, referred to CR, were capable of levels of walking fitness, achieved through challenging incremental test, comparable to the 25th–50th centiles of patients with conventional cardiovascular disease attending CR.^17^ Although the extent of walking fitness was greater by 42 m in the group with fewer comorbidities (≤3), a good mean distance of 240 m was achieved in the group having greater than three additional comorbidities. These findings help establish a positive picture in terms of physical fitness for those patients with the diagnosis of HF and willingness to engage in rehabilitation.

Using mean age of the study population (64.5±12.7) as a reference value, being older by 1 year was significantly associated with reduced walking distances in the region of 4.9 m for each year above mean age. This relationship is not new as it is evident in the conventional CR patient populations^16 17^; however, the ability to quantify the extent of loss, with increasing age, represents a novel finding in patients with HF.

After accounting for comorbidities gender continues to play a significant part in determining walking fitness with female patients having a 42 m reduced walking distance. The CR literature and routine practice data, captured in national reports,^3^ highlight that around 80% of patients with HF are missing out on CR and of those who do attend around one-third have a recorded physical fitness assessment.^3 11^ National clinical guidance worldwide recommends assessment of physical fitness prior to starting CR^6 7 13^ yet two-thirds of patients in the UK are not assessed. In terms of HF the lack of physical assessment may not purely be the fault of programmes and could, in part, be explained by the incapacity of patients or in some cases a perceived incapacity to carry out a walk test especially an incremental test. Our research has produced reference values to help clarify physical fitness expectations for patients with HF attending CR and for clinicians to aid goal setting at the start of CR (table 4).

A knowledge of age and gender differences in walking fitness aligned with fitness determining comorbidities such as COPD and depression, as shown in table 4, is considered useful in clarifying HF-specific physical fitness expectations for CR programme staff, patients and carers.

The presence of COPD in the form of chronic bronchitis or emphysema is significant in predicting fitness in patients with HF resulting in a 39 m reduction walking distance. COPD is dominated by the symptom of breathlessness, at rest, and exacerbated by physical effort which on top of the diagnosis of HF possibly explains such lower levels of fitness.^18^ The severity of HF as measured by NYHA did not reach significance as a determinant which might be explained by the analysis accounting for COPD. Within the NYHA class groups the proportion with COPD was 4% for class 1, 5% for class 2, 11% for class 3% and 31% for class 4. The inter-relation between HF and COPD is becoming increasingly important clinically leading to a call for a service provision aimed at managing breathlessness in patients with these comorbidities.^18^

A new finding for patients with HF was that higher levels of depression, measured using HADS, were strongly associated with poorer walking fitness. This has not been investigated previously in HF; however, our findings concur with a robust systematic review in healthy and depressed adult populations where depression and physical fitness were found to be inversely correlated.^19^

Table 4 incorporates the impact of COPD and depression on walking fitness reference values for younger and older patients with the combination of HF+COPD or HF+depression. This is the first set of reference values to account for the presence of such variables alongside HF which should enable clinicians to better understand physical fitness differences and/or expectations of their patients.

Our findings have generated new knowledge to help guide clinical decisions about the suitability of patients with HF to take part in exercise-based CR. The study also produced a set of reference values aligned with age, gender, COPD and depression to aid the interpretation of walking fitness in patients with HF in the hope that this new knowledge will help improve referral and uptake to CR.

### Limitations

Although the study investigated a large number of covariates, we were unable to account for medications such as diuretics and beta-blockers that may have accounted for some variation within the population in terms of weight gain and walking fitness, respectively.

Concerning our subanalysis of HF severity using NYHA there was a potential reporting bias through missing data. Although we cannot rule this out our analysis of the population with and without a recorded NYHA classification showed no significant differences for age and gender.

The mean age of patients recruited was 64.5 (SD 12.7) years which is below that seen in the general HF population. Our approach to ISWT reference values utilises the median distribution above and below 67 years which better reflects the full range of patients.

## Conclusion

The study concludes that patient age, gender, ethnicity, physical activity and depression status, along with the presence of COPD as comorbidity, were significant factors in predicting walking fitness in patients with HF. This is the first study to clarify the extent by which patient characteristics determine walking fitness in patients with HF and these findings have the potential to aid clinician understanding of the levels of fitness patients with HF can achieve. The study produced a novel set of reference values, aligned with age, gender, COPD and depression, to aid the interpretation of walking fitness by clinicians and patients. Through dissemination of these findings we believe this knowledge has potential to improve confidence in clinicians making a referral to CR and lead to a greater number of patients undergoing a physical fitness assessment thus enabling an appropriate exercise prescription.
