# Correction: *Determinants of walking fitness in patients with heart failure attending cardiac rehabilitation*

**DOI:** 10.1136/openhrt-2018-000866corr1

**Published:** 2019-05-10

Doherty P, Harrison AS, Hossain R. Determinants of walking fitness in patients with heart failure attending cardiac rehabilitation. *Open Heart* 2019;6:e000866. doi: 10.1136/openhrt-2018-000866

The authors want to alert readers to the following errors were identified in the published version.

At page 6 Table 4, the values under first data column 'mean distance' is incorrect and needs to be removed (ie, to remove the values 268, 194, 237 and 181 from the table). And shift the four rows to the left which will leave the column 'Count’ empty and these should be filled with the following values: 51, 59, 95 and 52.

The Table 4 should read as below:

**Table 4:** Incremental shuttle walk test reference values for heart failure (HF) patients split by age, gender and presence chronic obstructive pulmonary disease (COPD) and depression

Heart failure (HF) +comorbidity category	Age & Gender		Incremental shuttle walk test (ISWT)						Count
			Mean distance (metres)	SD	Percentile 05	Percentile 25	Percentile 75	Percentile 95	
HF only	<67 years	Male	338	180	70	200	460	630	443
		Female	285	145	70	190	350	520	190
	67+years	Male	243	138	60	140	330	480	434
		Female	184	109	40	100	250	390	211
HF +COPD	<67 years		237	133	40	120	330	430	51
	67+years		197	120	40	90	270	470	59
HF +Depression	<67 years		261	143	30	150	340	520	95
	67+years		233	107	70	155	295	420	52

